# Quality of Life, Depression, and Anxiety in Patients Undergoing Renal Replacement Therapies in Saudi Arabia

**DOI:** 10.1155/2022/7756586

**Published:** 2022-03-29

**Authors:** Emad Adel Shdaifat

**Affiliations:** Department of Community Health Nursing, College of Nursing, Imam Abdulrahman Bin Faisal University, P.O. Box 1982, City Dammam, Saudi Arabia

## Abstract

Despite improvements in renal replacement therapy (RRT) for end-stage renal disease (ESRD), it continues to have serious negative impacts on quality of life (QOL) and emotional status. This study determines the association between demographic characteristics and the negative emotional states of depression, anxiety, and stress in Saudi Arabia. A comparative cross-sectional study was conducted in the Eastern Region of Saudi Arabia. Convenience sampling was used to recruit participants comprising hemodialysis (HD), peritoneal dialysis (PD), and kidney transplantation (Tx) patients. They completed the Short Form-36 Survey and the Depression, Anxiety, Stress Scale (DASS). The physical component summary (PCS) score was similar for HD (41.7) and PD (41.5), but higher among Tx (45.2). The mental component summary (MCS) score was similar between HD (48.0) and Tx (48.8), but lower in PD (42.3). The majority of patients in all groups had normal levels of depression, anxiety, and stress. Overall, the study found that PCS was higher among Tx patients compared to HD and PD, while MCS was higher among HD and Tx patients than PD patients. Most patients' levels of depression, anxiety, and stress were within the normal range. Those findings will provide policymakers and health managers with the significant factors which can affect the QOL of dialysis and Tx patients.

## 1. Introduction

Chronic kidney disease (CKD) caused 1.2 million deaths in 2020 and was the twelfth leading cause of death worldwide [1]. CKD is a very stressful disease which causes many complications [2]. ESRD limits the choice of patients to RRT, including dialysis (hemodialysis or peritoneal dialysis) or kidney transplantation [3]. Consequently, the available modalities of these treatment options entail many significant changes that undermine patients' QOL [4]. According to Chen et al. [5], patients are the best people to evaluate their own QOL.

Quality of life is a substantial indicator of welfare, which helps in health planning by shaping care priorities and the effectiveness of healthcare interventions [6]. QOL is a complicated and multifaceted concept that affects and is shaped by health outcomes. It also encompasses broader concepts such as physical health and mental, psychological, spiritual, and social wellbeing, as well as self-image and self-determination, all of which are intrinsic to QOL and which affect health outcomes [7, 8]. The World Health Organization (WHO) defines QOL as follows:

“An individual's perception of their position in life in the context of the culture and value systems in which they live and relate to their goals, expectations; standards and concern and this include six domains; physical health, psychological status, level of independence, social relationship, environmental features, and spiritual concern” [9].

QOL research is important in evaluating the outcome of any intervention in chronic diseases. ESRD effectively incurs disability for patients and fundamentally changes their lives, leading to reduced QOL [10]. It is a measured attempt to assess the direct impact of medical intervention on patients' ability to function well in their daily lives [11].

Since dialysis and Tx are life-prolonging and not curative treatments, they affect many patients' psychological health status. Depression and anxiety are the most commonly reported mental problems, and psychological factors have a negative influence on patients' QOL [12–15]. A systematic review and meta-analysis by Palmer et al. [16] found that depression affects approximately a quarter of adults with CKD. Many scholars agreed that the prevalence of depression and anxiety among ESRD patients was higher than in the general population, as found in a single center in Saudi Arabia [14, 17]. Shirazian et al. [18] in their narrative review study found that the prevalence of depression is up to four times higher in patients with ESRD compared with the general population, and up to three times higher than in patients with other chronic illnesses.

AlDukhayel [19] carried out a study in Riyadh city in a single center and reported a high depression prevalence among PD and HD patients, at 98.5% and 83.5%, respectively. In India, in a single center, the researcher reported varying degrees of HD patient depression prevalence, e.g., 61% [20], 68% for two centers in Brazil [21], and a relatively lower rate of 23.3%, the results from two centers in Makkah, Saudi Arabia [22]. In Panama, a single center data reported 21.1% anxiety among such patients. However, depression prevalence among kidney transplant patients has been found to be relatively low, varying from 11.8% [23] to 25.7% according to the results from a meta-analysis study [16].

In a cohort study in Japan which is part from the Dialysis Outcomes and Practice Patterns Study (DOPPS), the study found that QOL among dialysis patients is lower than that of patients who undergo kidney Tx [24]. According to a multicenter study in Pakistan, there are many factors that affect the QOL of HD patients, such as age, gender, BMI, employment, educational level, income level, and other variables that are significantly correlated with QOL in general [25, 26]. The results from a single center in Riyadh, Saudi Arabia found that the mean score of QOL among Saudis on HD was 54.2 for MCS and 52.7 for PCS [27].

QOL is an important outcome measure in the assessment of chronic diseases. It measures physical and social functioning, and perceived physical and mental well-being. The importance of QOL in ESRD patients is manifest in the clinical outcome of the disease and its treatments. Moreover, evaluating the demographic characteristics of patients can help health practitioners and planners to improve management for ESRD patients and their treatment, to improve their QOL. There may be significant limitations on patients' QOL. Most studies on QOL and ESRD have been done abroad, and QOL studies on Saudi patients are limited. To address these research gaps, the current study aimed to do the following:

1. Determine the level of QOL for ESRD patients undergoing dialysis or kidney Tx in Saudi Arabia
2. Discover the prevalence of negative emotional states of depression, anxiety, and stress for ESRD patients undergoing dialysis or kidney Tx in Saudi Arabia
3. Identify the association between QOL, DASS, and demographic characteristics

## 2. Materials and Methods

A comparative cross-sectional study was carried out in the Eastern Region of Saudi Arabia. There were 21,068 patients diagnosed with ESRD in 2019 in Saudi Arabia, of whom 19,522 were treated with HD, while the remainder (1546 patients) received PD. In the eastern region in 2019, 2,416 patients received HD and 300 received PD, while there were 204 transplant patients [28]. Patients on PD treatment used automated peritoneal dialysis (APD).

The population sample consisted of all patients diagnosed as ESRD and treated with HD, PD, or Tx. Convenience sampling was utilized, with the following inclusion criteria: all participants aged 18 years or older, on dialysis or Tx for more than 3 months prior to the survey date (to allow patients to recover and stabilize their medical status, and return to activities of daily living) [29, 30].

The data were collected form HD patients in their unit which has 105 machines utilized by 218 patient who frequently on hemodialysis treatment two or three times per week. The unit is part from a public central hospital. In the PD unit which includes 33 patients, the patients are having their PD procedure through using an APD machine at home, and they should have frequent visits to the unit for medical evaluation and to collect their supplies and medicine. The Tx center is part of a specialist hospital and provides kidney transplants to the region with about 200 surgeries annually. The data were collected by face-to-face interviews while patients were on dialysis or waiting for their routine visit to collect medicine or blood test as a follow-up visit. The face-to-face interview was conducted to consider illiterate and weak patients. In addition, to give more explanation to them depend on their education level and mental health which will help in improve the quality of data. The minimum required sample size was calculated as 213 patients using *G∗*Power software. With an estimated effect size of 0.25, and alpha was 0.1 among three predictors for analysis of variance (ANOVA) [17].

The 36-Item Short Form Survey (SF-36) is a standard questionnaire used for many purposes. It is a short-form health survey with 36 questions, and it is one of the most widely used measures of health-related QOL [14], with a valid and reliable Arabic-language version [31]. The SF-36 health survey is a generic outcome measure designed to examine a person's perceived health status. It is a self-reported questionnaire, which includes eight health concepts, including physical function, role limitation because of physical health problems, bodily pain, social function, general mental health, role limitation due to emotional problems, vitality (energy/fatigue), and general health perception [32].

The Arabic version of the Depression, Anxiety, and Stress Scale (DASS) was designed to measure the level of the negative emotional states of depression, anxiety, and stress [33]. The DASS scale contains 42 items. It is a self-reported questionnaire designed to measure the severity of symptoms of depression and anxiety. The respondents were asked to indicate the presence of a symptom during the previous week. Each item is scored from 0 (absence of stress and depression during the last week) to 3 (a high level of depression and stress during the past week).

The study protocol was approved by the Institutional Review Board (IRB) at the university where the study was carried out. Participants' written permission was obtained by means of an informed consent form before data collection, including assurance that their participation was entirely voluntary and would not affect the care they received, and that their data would be maintained confidentially, available only to the research team. The data collection was started after ethical clearance was secured. SPSS was used to determine frequencies and percentages for demographic data and the DASS and the difference between groups. The mean and standard deviation (SD) were used to present QOL, and SF-36 software was used to calculate the score of QOL.

## 3. Results

The majority of the patients were male, married, educated to grade 9–12, and nonsmokers in the three studied treatment categories. Their mean ages were 49.1, 40.4, and 43.2 years old for HD, Tx, and PD patients, respectively. Dialysis hours per session were 4 for HD patients and 9 for PD patients. For Tx patients, the mean time since kidney transplantation surgery was 33.3 months, with a median of 22.5 months. The length of the period on dialysis for HD and PD was 44.7 and 24 months, respectively (Table 1).

QOL for PCS and MCS were calculated. The PCS score was similar for HD (41.7) and PD (41.5), but higher among Tx (45.2). The MCS score was similar between HD (48.0) and Tx (48.8), but lower in PD (42.3). On the DASS scale, for depression level, the majority of patients had a normal level of 57.1%, 73.9%, and 50.0% for HD, Tx, and PD, respectively. Anxiety was normal among the majority of patients in HD (60.0%), Tx (72.8%), and PD (55.0%). Additionally, stress was within the normal range for all categories of patients: HD (55.0%), Tx (62.0%), and PD (40.0%) (Table 2).

In HD, higher PCS scores were reported for males (*p* value 0.004); patients with education levels G9–12 (compared to illiterate ones) (post hoc, *p*=0.047) and higher than BSc (post hoc, *p*=0.025); and employed patients (compared to retirees) (post hoc, *p*=0.013). For Tx, higher PCS was reported for unmarried patients (*p*=0.05), patients with G9–12 (post hoc, *p*=0.033), and smokers (*p*=0.048). For PD, the PCS score was also higher among smokers (*p*=0.022) (Table 3).

## 4. Discussion

This current study found that PCS was higher in Tx patients compared to their HD and PD counterparts, while MCS was higher among HD and Tx patients compared with those receiving PD. In HD, PCS was higher among males, patients with an education level of G9–12, and those in employment. For Tx, PCS was higher among unmarried patients, those with G9–12, and smokers. For PD, the PCS score was higher among smokers. In the DASS scale, most patients' levels of depression, anxiety, and stress were within the normal range.

This study confirmed that PCS was higher in Tx patients compared to those receiving HD and PD. Also, MCS was higher among HD and Tx patients compared with PD patients. These results corroborate previous studies in a single center in Sichuan Province, China, and in a multi-center in China and Trinidad and Tobago [34–36]. A 10-year follow-up cohort study in Brazil involving ten dialysis centers reported that patients who shifted from hemodialysis or peritoneal dialysis to transplantation modalities gained better PCS and MCS scores [4]. Similarly, in Poland, a prospective study on a single-center evaluating QOL before Tx and one year after found that QOL improved after Tx for patients who were previously receiving PD or HD [37]. Additionally, a four-year multicountry cohort study with 1,685 patients comprising patients with CKD (not on dialysis), patients on dialysis (HD or PD), and transplanted patients found that QOL was highest among Tx and then CKD patients, and was worst among receivers of dialysis [38]. However, a systematic review reported that most studies found no significant differences in QOL among patients with ESRD receiving HD or PD treatment modalities [39]. On the other hand, a study in a single center in Sichuan Province, China, by Zhang et al. [34] found that the peritoneal dialysis group had higher scores of PCS and MCS than those of the hemodialysis group. For HD patients, the average level of QOL was high among CCG countries including KSA for both domains of PCS and MCS [40].

Although no single treatment provides a complete cure for the studied factors, transplant patients seem to function more normally, and one of the benefits of transplantation is to offer a state of health similar to what patients had before the onset of the disease [37]. A possible explanation for this might be that after transplantation, patients can excrete metabolic waste without mechanical intervention, and their improved nutritional status will reflect positively on their physical and mental status. Moreover, the freedom from schedule sessions will offer more chances to be involved in normal life activities that improve wellbeing and QOL [34]. On the other hand, HD patients undergo painful procedures such as needling and suffer from side effects of dialysis, such as hypotension and heart problems. Also, HD patient machine dependence and modified body fluid balance can affect their mentality, while PD does not include painful procedures, thus patient status is relatively stable [34].

The results of this study showed that in Tx patients, PCS was higher among the unmarried, patients with G9–12, and smokers. In HD, PCS was higher among males, patients with educational levels G9–12, and those in employment. For PD, the PCS score was higher among smokers. Correspondingly, the results from a multinational study reported that factors associated with reduced QOL included being female, having a lower educational level, and being single [38]. In Jordan, the data from one center found that HD patients' PCS was associated with age, gender, marital status, and depression and MCS was significantly associated with age, gender, marital status, education, and depression [41]. In France, data from a multi-center found that PCS was lower among older and female Tx patients [42].

A multinational study found that married patients experienced better QOL [38]. Furthermore, in Brazil, the results from two centers found that smoking negatively affected the perceptions of QOL among HD patients [43], although another study found no significant relation between Tx and HD in relation to smoking status in the Netherlands [44].

The justification of worse outcomes for women may be that they are a vulnerable group with chronic disease since they have different psychosocial perspectives on life compared with men according to multinational study [38]. Despite smoking being a well-known predictor of numerous diseases, with high levels of mortality, severe health problems, and decreased QOL, some studies found that smoking was used as a means to avoid being alone, a source of solace, and a way to reduce pain (i.e., some patients may assume that smoking is a way of dealing with CKD and its outcomes). The study was carried out in Brazil [43]. Because of the nature of kidney disease, a relatively higher level of education will allow patients to play a more proactive role in managing their own health status as point out in a multinational study [38]. Furthermore, education level is very crucial to improve QOL. According to the narrative review by Aguiar et al. [45], patient capability to engage with health knowledge and services is correlated with improved mental health and QOL, and dialysis patients with better health literacy had better QOL and lower levels of anxiety and depression.

With regard to the DASS scale, most patients' levels of depression, anxiety, and stress were found to be within the normal range, in line with a study by [46], who found that the majority of dialysis patients had normal psychological status. This is consistent with results from Singapore from one center by Lai et al. [47], who found that Tx patients have a normal range of anxiety and depression. Conversely, a multinational study by Krishnan et al. [38] found that transplant patients have lower levels of anxiety and depression compared to dialysis patients. Additionally, in Iran, a study by Shafipour et al. [48] including three centers found that the majority of HD patients had severe depression, anxiety, and stress levels, which may be attributable to PD being associated with increased autonomy and flexibility in lifestyle and fewer social boundaries (e.g., more flexibility to travel, eat, and drink). Dialysis during sleeping time also improves QOL [46]. For HD and Tx patients, the likely explanation for low DASS scores may be due to the age of the patients, who were mainly in the middle-aged group. Another possible reason was that the majority of patients in the present study were male, and females generally have poorer wellbeing and QOL.

Finally, the current study had several important limitations that need to be considered. The sampling setting limits the generalizability of the findings to other areas or settings. Additionally, the cross-sectional design does not evaluate changes in QOL over time. Furthermore, the limited number of PD participants does not help us in carrying out regression analysis for this group.

## 5. Conclusion

This study was designed to determine the level of QOL in relation to the negative emotional states of depression, anxiety, and stress for ESRD patients undergoing dialysis or kidney Tx.

This study found that PCS was higher in Tx compared to HD and PD, while MCS was higher among HD and Tx than for PD patients. In HD, PCS was higher among males, patient with an education level of G9–12, and working patients. For Tx, PCS was higher among unmarried, G9–12, and smoking patients. For PD, the PCS score was higher among smokers. In the DASS scale, most patients' levels of depression, anxiety, and stress were within the normal range.

These findings have significant implications for understanding the QOL among patients on ESRD. It is expected that findings from this study will provide health care managers and policy developers with significant information about the impact of dialysis and its treatment on patients' QOL and help identify patients' needs, as well as particularly vulnerable patient groups. An implication of this study is the possibility that the findings provide direction for hospital decision makers on the areas that could be improved to ensure better QOL among patients. This research allows managers to better understand the relation between QOL and other variables. This will enhance awareness of pertinent risk outlines, to help administrators address such factors.

The present study extends knowledge about QOL among patients on ESRD. A key strength of the present study was the inclusion of the three modalities at the same time for the same area, to be a basis for comparison. More research is required to develop a deeper understanding of the complexity of QOL in ESRD and its longitudinal changes over time.

## Figures and Tables

**Table 1:** Demographic characteristics of patients.

		HD		TX		PD	
		No.	%	No.	%	No.	%
Gender	Male	80	76.2%	58	63.0%	11	55.0%
	Female	25	23.8%	34	37.0%	9	45.0%

Marital status	Unmarried	43	41.0%	31	33.7%	9	45.0%
	Married	62	59.0%	61	66.3%	11	55.0%

Education level	Illiterate	20	19.0%	4	4.3%	2	10.0%
	G1–8	20	19.0%	18	19.6%	4	20.0%
	G9–12	39	37.1%	36	39.1%	7	35.0%
	Diploma	11	10.5%	10	10.9%	4	20.0%
	BSc and above	15	14.3%	24	26.1%	3	15.0%

Employment status	Working	21	20.0%	33	36.7%	11	55.0%
	Not working	61	58.1%	39	42.4%	6	30.0%
	Retired	23	21.9%	20	21.7%	3	15.0%

Smoking	Yes	26	24.8%	6	6.5%	3	15.0%
	No	79	75.2%	86	93.5%	17	85.0%

Nationality	Saudi	50	47.6%	89	96.7%	17	85.0%
	Non-Saudi	55	52.4%	3	3.3%	3	15.0%
		Mean (SD)	Median (IQR)	Mean (SD)	Median (IQR)	Mean (SD)	Median (IQR)

Age		49.1 (16.5)	50.0 (24.5)	40.4 (14.3)	36.0 (23.5)	43.2 (13.4)	40.5 (24.5)
Time since start the modality		44.7 (43.5)	24.0 (51.0)	33.3 (35.1)	22.5 (40.8)	35.5 (32.6)	24.0 (51.0)
Dialysis hour		4.0 (0.2)	4.0 (0.0)	-	-	9.1 (1.9)	8.5 (1.8)

**Table 2:** Mean score and frequency for QOL and DASS domains.

			HD	Tx	PD
			Mean (SD)	Mean (SD)	Mean (SD)
QOL	PCS		41.7 (10.4)	45.2 (8.6)	41.5 (8.1)
	MCS		48.0 (9.0)	48.8 (9.4)	42.3 (10.2)

			Frequency (%)	Frequency (%)	Frequency (%)
DASS	Depression	Normal	60 (57.1)	68 (73.9)	10 (50.0)
		Mild	24 (22.9)	12 (13)	3 (150)
		Moderate	9 (8.6)	9 (9.8)	3 (15.0)
		Sever	6 (5.7)	2 (2.2)	3 (15.0)
		Extremely sever	6 (5.7)	1 (1.1)	1 (5.0)
	Anxiety	Normal	63 (60.0)	67 (72.8)	11 (55.0)
		Mild	13 (12.4)	11 (12.0)	2 (10.0)
		Moderate	11 (10.5)	9 (9.8)	6 (30.0)
		Sever	8 (7.6)	4 (4.3)	0 (0)
		Extremely sever	10 (9.5)	1 (1.1)	1 (5.0)
	Stress	Normal	58 (55.2)	57 (62.0)	8 (40.0)
		Mild	21 (20.0)	5 (5.4)	2 (10.0)
		Moderate	12 (11.4)	17 (18.5)	3 (15.0)
		Sever	11 (10.5)	10 (10.9)	1 (5.0)
		Extremely sever	3 (2.9)	3 (3.3)	6 (30.0)

**Table 3:** The difference between QOL and Demographic variables.

		HD				TX				PD			
		PCS		MCS		PCS		MCS		PCS		MCS	
		Mean (SD)	*P* value	Mean (SD)	*P* value	Mean (SD)	*P* value	Mean (SD)	*P* value	Mean (SD)	*P* value	Mean (SD)	*P* value
Gender	Male	43.7 (9.8)	0.004^*∗*^	48.8 (8.2)	0.086	45.6 (8.8)	0.513	49.8 (9.8)	0.181	42.6 (10.0)	0.511	44.1 (10.1)	0.391
	Female	36.6 (12.8)		45.3 (10.7)		44.4 (8.3)		47.1 (8.7)		40.0 (5.2)		40.0 (10.4)	

Marital status	Unmarried	43.4 (10.4)	0.277	49.3 (8.2)	0.190	47.7 (8.3)	0.050^*∗*^	48.4 (8.3)	0.745	42.0 (8.4)	0.807	40.3 (5.2)	0.449
	Married	41.0 (11.3)		47.0 (9.4)		43.9 (8.5)		49.0 (10.0)		41.0 (8.3)		43.9 (13.0)	

Education level	Illiterate	39.3 (10.7)	0.004^*∗*^	47.2 (10.3)	0.455	39.0 (10.9)	0.031^*∗*^	46.3 (13.8)	0.361	34.5 (4.6)	0.683	49.5 (21.6)	0.762
	G1–8	39.8 (12.0)		48.0 (8.5)		40.8 (7.8)		49.2 (9.2)		40.1 (3.7)		43.7 (9.9)	
	G9–12	47.3 (8.3)		49.7 (7.8)		47.8 (6.3)		46.7 (8.8)		42.9 (9.9)		39.3 (8.3)	
	Diploma	37.9 (10.6)		47.8 (8.8)		44.5 (7.6)		50.9 (8.7)		44.7 (7.4)		40.3 (7.4)	
	BSc and more	37.7 (12.2)		44.6 (10.6)		45.8 (10.8)		51.3 (10.0)		40.2 (11.6)		45.2 (14.4)	

Employment status	Working	45.8 (7.4)	0.014^*∗*^	50.6 (4.1)	0.315	45.3 (7.5)	0.669	51.0 (9.4)	0.175	43.4 (9.8)	0.461	43.2 (9.0)	0.899
	Not working	42.7 (10.2)		47.2 (9.4)		45.8 (9.0)		48.3 (9.5)		38.1 (4.6)		40.7 (12.4)	
	Retired	36.6 (13.8)		47.5 (10.8)		43.7 (8.6)		46.2 (8.9)		40.8 (6.1)		42.0 (13.5)	

Smoking	Yes	44.2 (8.2)	0.150	47.4 (7.9)	0.726	51.9 (9.7)	0.048^*∗*^	50.2 (10.1)	0.720	51.0 (3.5)	0.022^*∗*^	45.0 (8.0)	0.619
	No	41.2 (11.7)		48.1 (9.3)		44.7 (8.4)		48.7 (9.4)		39.8 (7.5)		41.8 (10.6)	

Nationality	Saudi	41.8 (11.1)	0.861	49.2 (8.6)	0.164	45.2 (8.7)	0.915	48.6 (9.2)	0.357	41.8 (8.3)	0.707	42.7 (10.8)	0.671
	Non-Saudi	42.2 (10.9)		46.8 (9.2)		45.7 (3.0)		53.8 (16.4)		39.8 (8.1)		39.9 (6.0)	

^*∗*^Significant.

## Data Availability

The data that support the findings of this study are available on request from the corresponding author.
